# Functions and mechanisms of the GPCR adaptor protein Norbin

**DOI:** 10.1042/BST20221349

**Published:** 2023-07-28

**Authors:** Stephen A. Chetwynd, Simon Andrews, Sarah Inglesfield, Christine Delon, Nicholas T. Ktistakis, Heidi C. E. Welch

**Affiliations:** 1 Signalling Programme, Babraham Institute, Cambridge, U.K.; 2 Bioinformatics Facility, Babraham Institute, Cambridge, U.K.

**Keywords:** GPCR, NCDN, neurochondrin, Norbin, P-Rex1, Rac

## Abstract

Norbin (Neurochondrin, NCDN) is a highly conserved 79 kDa adaptor protein that was first identified more than a quarter of a century ago as a gene up-regulated in rat hippocampus upon induction of long-term potentiation. Most research has focussed on the role of Norbin in the nervous system, where the protein is highly expressed. Norbin regulates neuronal morphology and synaptic plasticity, and is essential for normal brain development and homeostasis. Dysregulation of Norbin is linked to a variety of neurological conditions. Recently, Norbin was shown to be expressed in myeloid cells as well as neurons. Myeloid-cell specific deletion revealed an important role of Norbin as a suppressor of neutrophil-derived innate immunity. Norbin limits the ability of neutrophils to clear bacterial infections by curbing the responsiveness of these cells to inflammatory and infectious stimuli. Mechanistically, Norbin regulates cell responses through binding to its interactors, in particular to a wide range of G protein-coupled receptors (GPCRs). Norbin association with GPCRs controls GPCR trafficking and signalling. Other important Norbin interactors are the Rac guanine-nucleotide exchange factor P-Rex1 and protein kinase A. Downstream signalling pathways regulated by Norbin include ERK, Ca^2+^ and the small GTPase Rac. Here, we review the current understanding of Norbin structure, expression and its roles in health and disease. We also explore Norbin signalling through its interactors, with a particular focus on GPCR trafficking and signalling. Finally, we discuss avenues that could be pursued in the future to increase our understanding of Norbin biology.

## Introduction

Norbin is a highly conserved adaptor protein first identified in 1997 [1]. Early on, Norbin was considered almost exclusively neuronal and was mainly studied for its important roles in neuronal morphology and synaptic plasticity, which were reviewed previously [2]. However, over recent years, Norbin was also found in myeloid cells, acting as an immune suppressor. We review here the physiological and pathophysiological roles of Norbin, and the mechanisms through which it acts, with particular emphasis on recent findings.

## Norbin evolution, structure and expression

### Evolution of the Norbin gene

**Figure 1.**
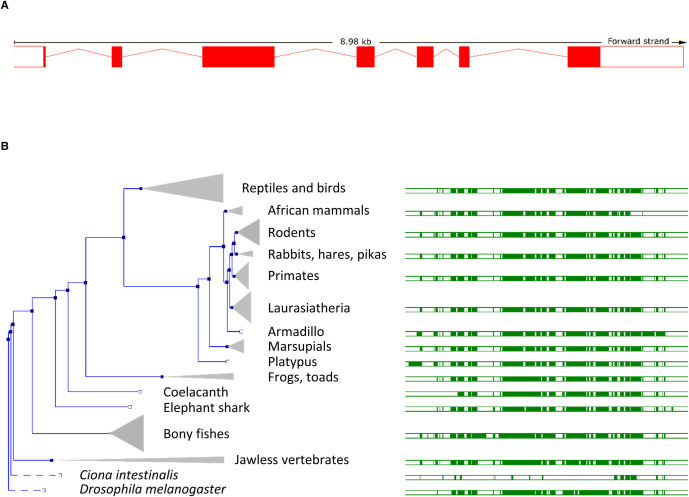
Norbin orthologs in animals. (**A**) Norbin gene structure highlighting its 7 exons (red boxes) and introns (red lines). In humans, *NCDN* is located on chromosome 4: 126 637 543–126 647 202. (**B**) Norbin (*NCDN*) gene tree in animals (left) generated by the Ensembl gene orthology/paralogy prediction pipeline using the *Mus musculus Ncdn* gene (ENSMUSG00000028833) as the reference sequence. Norbin is highly conserved throughout vertebrates, and is also found in invertebrates such as *Drosophila melanogaster*. The homology with the sea squirt *Ciona intestinalis* is limited, there is only a short fragment of similarity in the C-terminus, and the gene seems to have been lost from worms, as there is no Norbin in *Caenorhabditis elegans*. In mammals, birds and most fish, a direct 1-to-1 ortholog of human Norbin exists*.* In some fish species, gene duplication events have occurred, such as in carp, where there are two full-length copies with ∼85% identity. Highly conserved blocks of genomic sequences are indicated with green boxes (right).

**Figure 2.**
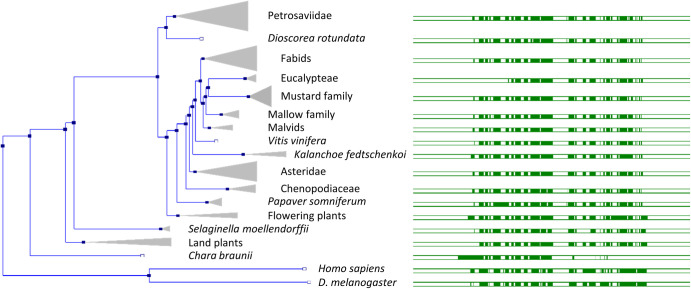
Norbin orthologs in plants. Norbin (*ncdn*) gene tree in plants (left), generated by the Ensembl gene orthology/paralogy prediction pipeline, using the *Arabidopsis thaliana ncdn* gene (AT4G32050) as the reference sequence. In plants, the protein is smaller than in animals (619 amino acids in *A. thaliana* compared with 729 in humans), but it is highly conserved between species. Highly conserved blocks of genomic sequences are indicated with green boxes (right). Human and *D. melanogaster* Norbin are included for reference.

**Figure 3.**
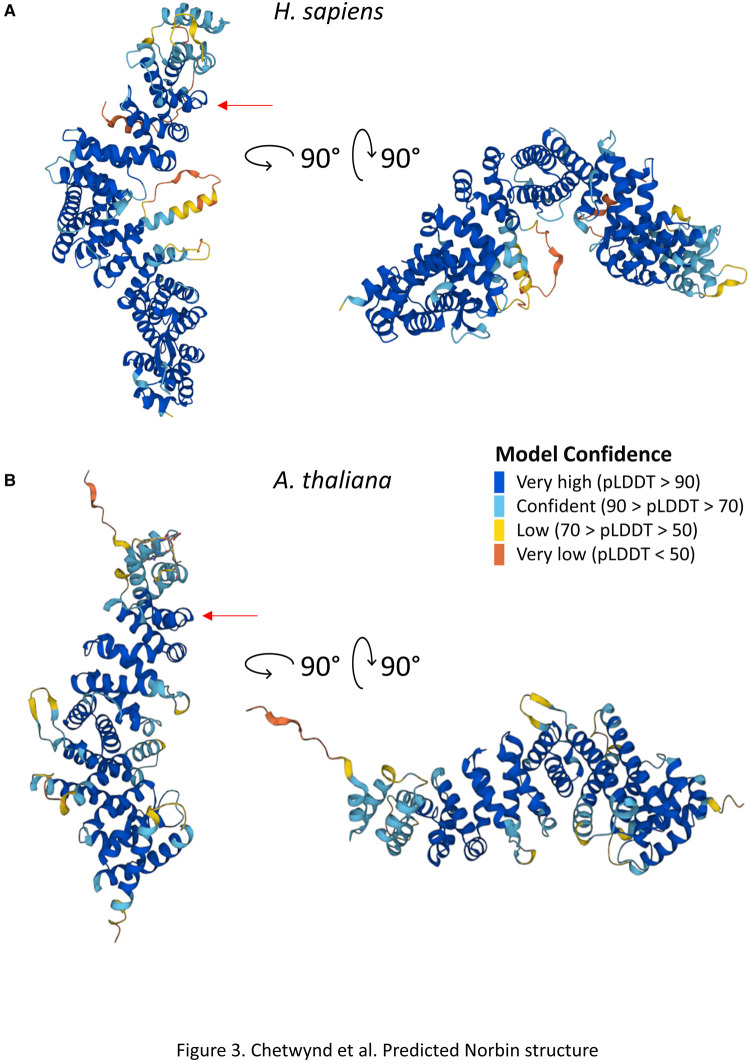
Predicted Norbin structure. Structural predictions for (**A**) human Norbin (UniProt: Q9UBB6) and (**B**) *A. thaliana* Norbin (UniProt: Q5E911) were obtained from the AlphaFold Protein Structure Database (alphafold.ebi.ac.uk). One highly conserved region between the two species is indicated with red arrows for reference. AlphaFold produces a per-residue confidence score (pLDDT) between 0 and 100. Regions below 50 pLDDT in isolation are considered unstructured.

Neurite-outgrowth-related protein from the rat brain (Norbin/Neurochondrin/NCDN) is an essential protein encoded by the *NCDN* gene, conserved from invertebrates to vertebrates, with high (98%) sequence identity among human, mouse and rat, and 47% similarity between human and *Drosophila melanogaster* (Figure 1). Our bioinformatic analysis shows Norbin expression even in plants, e.g. with 44% similarity between human and *Arabidopsis thaliana* (AT4G32050) (Figures 2 and 3). Norbin has never been studied in plants, except for a genome-wide association study in the Asian cotton (*Gossypium arboretum*) identifying an association between *ncdn* SNPs and salt tolerance [3]. It appears that Norbin was lost from worms, as it is absent from *Caenorhabditis elegans*.

### Norbin structure

Norbin is a 79 kDa protein without catalytic activity, homologies to other proteins, or known protein domains. Of note, InterPro designated a ‘neurochondrin domain’ spanning the length of the protein and describing the protein rather than a functional domain. Norbin is enriched in leucines (∼16%) and has interspersed low-complexity regions [4]. A structure has not been solved, although gel filtration indicated it is non-globular, and circular dichroism suggested an α-helical structure (∼65%) [5]. These observations are supported by *in silico* structures in AlphaFold (alphafold.ebi.ac.uk) predicting a curved structure like a boomerang, comprised of α-helices (∼67% of the protein) organising into units resembling HEAT repeats, flexible arrays of amphiphilic α-helices connected via a short linker sequence and arranged in an antiparallel conformation concealing their hydrophobic core (Figure 3) [2,6,7]. The curved conformation is thought important in protein–protein interactions, facilitating binding to structural features such as hydrophobic regions [8], and suggests Norbin may only adopt a fixed conformation in complex with other interactors.

### Norbin isoforms

**Figure 4.**
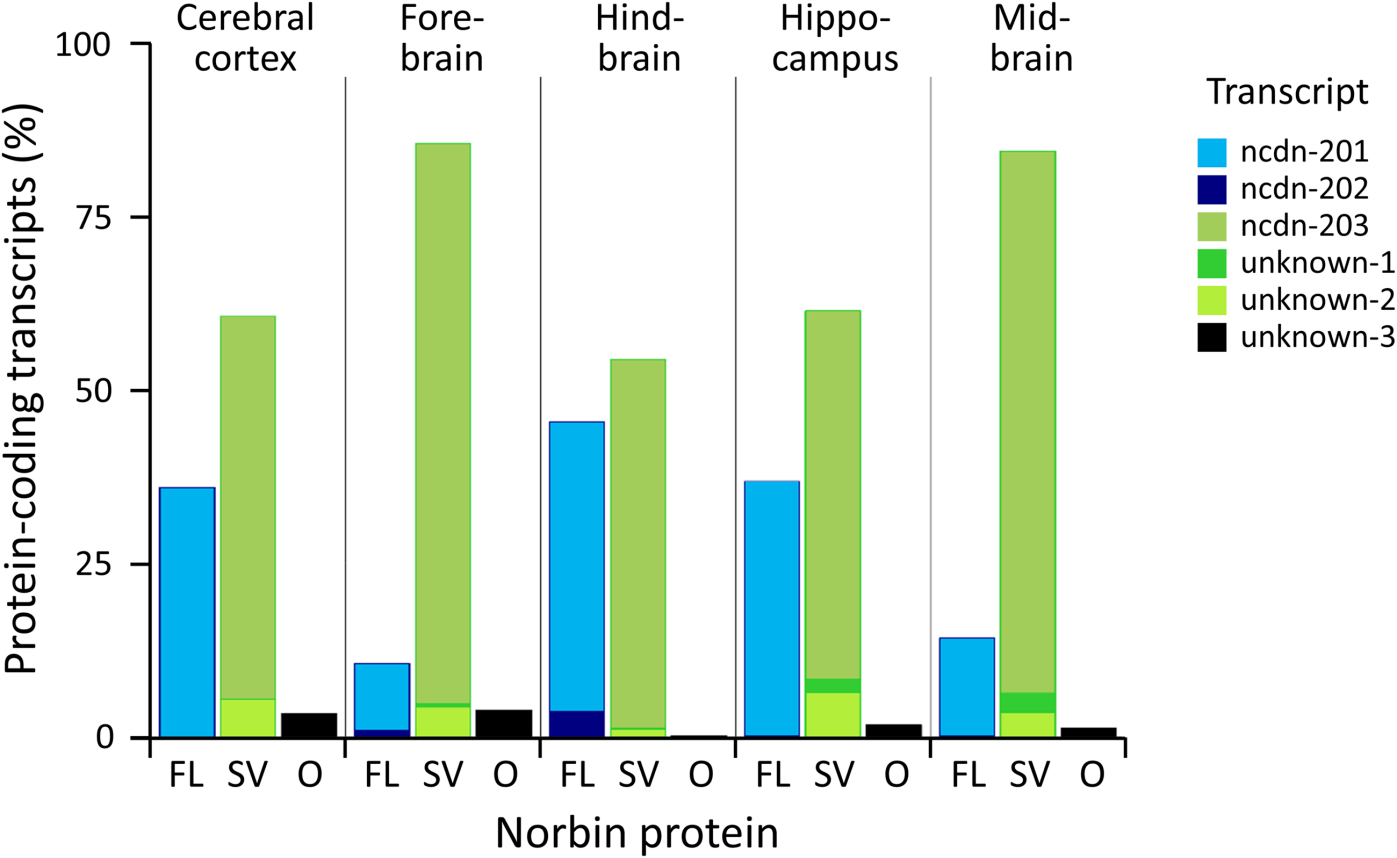
Distribution of protein-coding Norbin transcripts in brain regions of newborn mice. Bam files from the following ENCODE RNAseq datasets were analysed for the abundance of confirmed and putative protein-coding Norbin transcripts in various brain regions of newborn wild-type mice: ENCFF701BYJ, cerebral cortex. ENCFF760YQJ, forebrain. ENCFF983VMB, hindbrain, ENCFF165PCY, hippocampus. ENCFF398QVK, midbrain. Animals were aged between postnatal days 0 and 10, and were of both sexes. To analyse splice variants, intronic reads were quantitated in Seqmonk through read position probe generation over the Ncdn gene followed by exact overlap quantification, normalised to the total read count, with filtering to remove probes with quantitation <100. IDs of the known transcripts are according to Ensembl (v102). Transcript-associated protein forms are defined from Ensembl (where known) or as the amino acid sequence of the longest open reading frame generated by the transcript. The proteins that would result from these transcripts are full-length Norbin (FL, aa 1–729, blue shades), the known 712 aa splice variant (SV, aa 18–729, green shades), and a hypothetical 579 aa splice variant starting in exon 3 for which there is currently no experimental evidence (O, aa 151–729, black).

Two isoforms of Norbin were identified, the canonical full-length 729 aa isoform and a 712 aa isoform, N-terminally truncated through alternative splicing [4,9]. These isoforms are conserved between humans and mice, although only single bands presumed to be full-length were observed by northern and western blots [4,9]. To evaluate the abundance of Norbin variants, we analysed ENCODE RNAseq datasets of brain regions of newborn mice (Figure 4). This identified two transcripts (Ncdn-201 and Ncdn-202) which give rise to the 729 aa full-length protein (UniProt: Q9Z0E0-1) and one (Ncdn-203) encoding the 712 aa splice-variant (UniProt: Q9Z0E0-2). Furthermore, we found two previously undescribed transcripts that also encode the 712 aa splice variant and one that might encode a shorter hypothetical protein for which there is no experimental evidence. Ncdn-203 was predominant throughout the brain, particularly in the fore- and midbrain. Of the two RNAs encoding full-length Norbin, Ncdn-201 was most common. Extrapolating protein levels from transcript abundance, we propose the 712 aa splice variant may be the predominant Norbin protein in neonatal mouse brain, the full-length protein reaching comparable levels only in hindbrain. Isoform-specific antibodies or proteomics need to confirm this expression in future. Potentially, the two variants have different functions. Notably, the 712 aa splice-variant lacks cysteines C3 and C4, whose palmitoylation localises Norbin to early endosomes in hippocampal neurons [10].

### Norbin regulation

Little is known regarding Norbin regulation. A mechanism of intramolecular inhibition was proposed, as the isolated aa 1–100 of Norbin stimulated more neurite outgrowth in N2a cells than the full-length protein did, and bound Norbin aa 100–729 more robustly [11]. However, structural predictions do not support intramolecular interactions between the N- and C-termini. It was suggested that Norbin might dimerise [3]. Perhaps dimerisation, rather than intramolecular inhibition, serves to sequester inactive Norbin. Whether Norbin exists as a dimer, and if signalling alters its conformation or dimerisation state, remains to be demonstrated. The only other tangible evidence for Norbin regulation is related to subcellular localisation, through binding other proteins and lipids (see ‘Subcellular localisation’).

### Tissue distribution

Norbin was first identified as a gene up-regulated upon induction of long-term potentiation (LTP) in rat hippocampus [1]. This spurred extensive characterisation in the brain where Norbin is strongly expressed, particularly the hippocampus, cerebral cortex, cerebellum and amygdala [4,9,12,13]. Norbin is found in multiple types of neurons, localising to somatodendritic regions, but is absent from glial cells, as identified by the lack of expression in cells staining positive for glial fibrillary acidic protein [1,10,13–17]. Its neuronal expression is regulated by transcription factor FoxO3a [18].

Norbin expression is more widespread than first thought, detectable in the peripheral nervous system (sciatic nerve, brachial plexus), testes, ovaries, heart, lung, kidney and skeletal muscle [4,12,13]. In Drosophila, Norbin is expressed in fibrillar skeletal muscle, limiting sarcomere branching during myofibril formation, is required for the ability to fly [19], and is up-regulated during hyperactivity-induced myopathy [20]*.* Norbin is also expressed in bones (nucleus pulposus, osteoblasts, osteocytes, chondrocytes) and linked with resorption [9,21–23]. Recently, Norbin was shown to play an important role in the immune system, where it is expressed in the myeloid lineage (neutrophils and macrophages), as well as in the thymus (see below) [12,24].

### Subcellular localisation

Norbin is mostly cytosolic, in a range of cell types [10,12,13,15]. Co-expression with the guanine-nucleotide exchange factor (GEF) P-Rex1 in endothelial cells translocates both Norbin and P-Rex1 to the plasma membrane [12]. Furthermore, Norbin associates with G protein-coupled receptors (GPCRs) (see below), which also promotes plasma membrane localisation [25]. Norbin remains attached to the GPCR even after prolonged agonist stimulation, and potentially during receptor endocytosis [25]. Accordingly, Norbin associates with Rab5-positive endosomes in cortical and hippocampal neurons, and co-purifies with Rab5 upon co-expression in SH-SY5Y human neuroblastoma cells [10,15].

Norbin can be palmitoylated at cysteines C3 and C4, by palmitoyl-transferases DHHC 1, 3 and 10, its endosomal localisation depending on this palmitoylation [8]. Palmitoylation is a reversible post-translational modification, which can confer plasma-membrane association and trafficking between membrane compartments, sometimes in response to signalling [26]. However, regulation and consequences of Norbin palmitoylation remain unknown.

**Figure 5.**
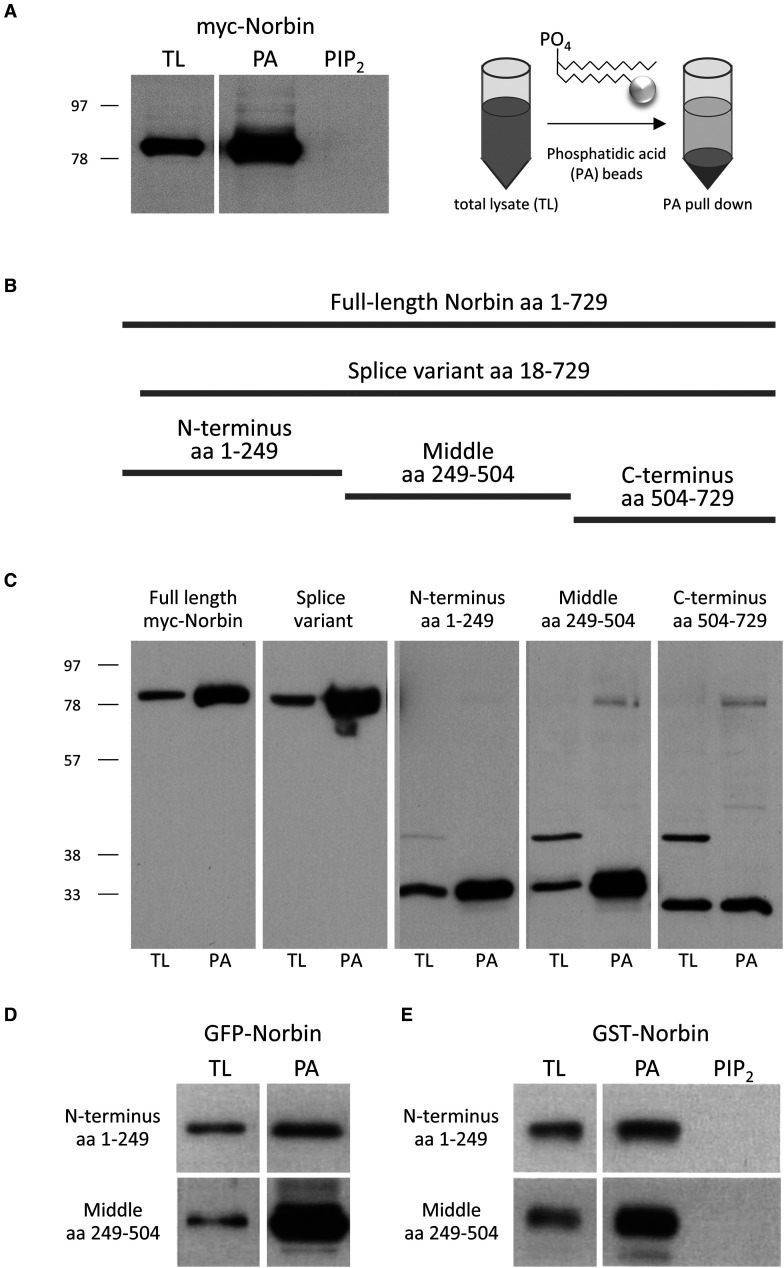
Norbin binding of phosphatidic acid. (**A**) Total lysate (TL) of COS-7 cells expressing full-length myc-tagged Norbin was subjected to pull-down with immobilised phosphatidic acid (PA) or control phosphatidylinositol (4,5)-diphosphate (PIP_2_) as described in [27] and was western blotted with myc antibody. One percent of the TL was loaded for comparison. (**B** and **C**) Myc-tagged full-length Norbin and the indicated mutants were subjected to PA pull down as in (**A**). (**D**) GFP-tagged N-terminal or internal fragments of Norbin were expressed in COS-7 cells and the TL subjected to PA pull down as in (**A**) and were western blotted with GFP antibody. (**E**) GST-tagged N-terminal or internal fragments of Norbin were expressed in *E. coli* and the TL was subjected to pull down with PA or PIP_2_ beads as in (**A**) and was western blotted with GST antibody. (**A**,**C**–**E**) Western blots shown are representative of three independent experiments.

Finally, Norbin interacts with phosphatidic acid (PA) [27], a phospholipid second messenger generated by phospholipase D and diacylglycerol kinases [28,29]. Using immobilised phosphatidic acid, we show that both full-length Norbin and the 712 aa splice variant bind PA (Figure 5A–C). Mutagenesis revealed that PA binds mainly to the middle portion, but also the N- and C-termini (Figure 5C–E). In contrast, Norbin does not bind phosphatidylinositol (4,5)-bisphosphate (PIP_2_) (Figure 5A,E). Hence, Norbin might associate with cell membranes in response to PA-generating signalling pathways.

Several studies proposed that Norbin may be secreted. Norbin was found in the conditioned medium of BW5147 metastatic T cell lymphoma cells [9], and in the extracellular uterine luminal fluid in cattle [30]. The frequent occurrence of Norbin autoantibodies in autoimmune-diseases (see below) may add further evidence. However, Norbin is largely cytosolic, any interactions with lipids and membrane proteins place it on the cytoplasmic face of membranes, and it has no signal sequences which would destine it for secretion. Rather, Norbin might escape cells upon their death, and as it is highly abundant in neurons, an appreciable amount could be found extracellularly upon neuronal death.

## Physiological roles of Norbin

### Norbin functions in the nervous system

Norbin was recognised early on for its functions in the nervous system. Whole-mouse knock-out is embryonic lethal [31], and conditional knock-out in parts of the nervous system results in spatial learning defects, epileptic seizures, impaired cognitive functions, and depression- or schizophrenia-like behaviours [31–33]. In neuronal cell lines, neurite outgrowth depends on Norbin [11,13,18], although mice with nervous system-wide deletion show no obviously defective neuronal morphology [32]. In mice with Norbin deficiency in postnatal forebrain, hippocampal synaptic plasticity is impaired [34], and hippocampal Norbin deficiency affects adult neurogenesis [33]. Neuronal Norbin expression can respond to extracellular cues. For example, Norbin was up-regulated in the amygdala upon exposure of rats to cat odours to induce fear [35], and in the brain of bees after olfactory training exercises [36].

### Norbin functions in the immune system

Recent work from our laboratory identified Norbin as a suppressor of innate immunity. Mice with Norbin deficiency in the myeloid lineage (Ncdn^Δmye^) had 10-fold elevated immunity during pulmonary pneumococcal infection and septic peritonitis [24]. Using immune-cell depletion, we showed that Norbin deficiency in neutrophils was responsible for this elevated immunity, whereas Norbin deficiency in macrophages had no effect [24]. During pneumococcal infection, overall leukocyte recruitment was normal, but neutrophils migrated more rapidly into the infected alveolar airspace [24]. The responsiveness of isolated Ncdn^Δmye^ neutrophils to a range of stimuli was increased, with enhanced degranulation, phagocytosis, production of reactive oxygen species (ROS) and neutrophil extracellular traps (NETs), and ROS-dependent killing of bacteria [24]. Neutrophils are tightly regulated, their excessive responses can exacerbate inflammatory diseases such as rheumatoid arthritis [37], but despite Norbin functioning as an immune-suppressor, autoinflammation was not detected in Ncdn^Δmye^ mice [24]. Of note, our recent evaluation of the neutrophil proteomes from young and old mice showed no age-related difference in Norbin expression, suggesting no obvious role in inflamm-aging, a state of chronic, low-grade sterile inflammation associated with aging [38]. Furthermore, in lymphoma-derived MSB-1 cells, Norbin was shown to indirectly affect the phosphorylation of nuclear factor of activated T cells (NFAT), and may thus play a role in transcription [39]. It would be interesting to assess Norbin in chronic inflammation and in different types of immune cells.

## Mechanisms of Norbin function

The mechanisms through which Norbin carries out its functions are incompletely understood. The best-described mechanism is through its constitutive, direct interaction with GPCRs, which regulates GPCR trafficking and/or signalling. Other mechanisms include the regulation of Rac GEFs and protein kinase A (PKA).

### Norbin interaction with GPCRs

**Figure 6.**
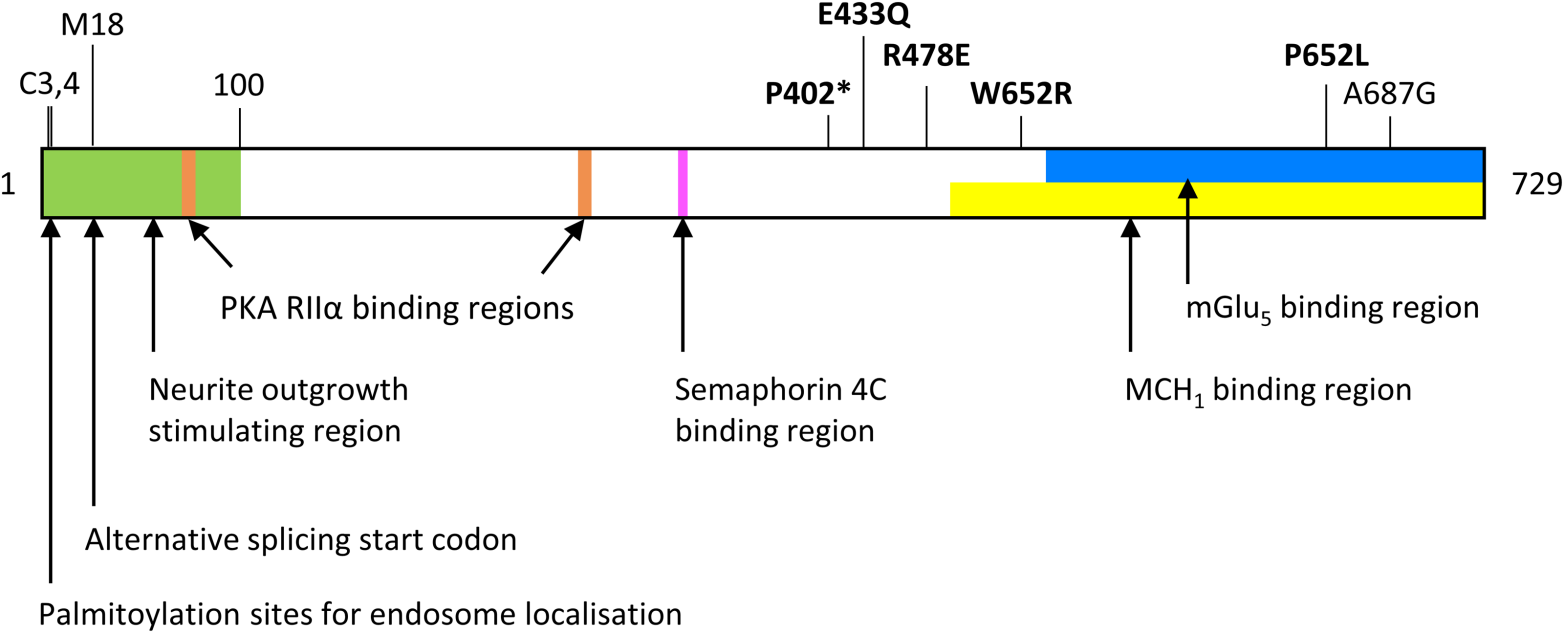
Map of Norbin residues with known function. Binding regions of mGlu_5_ (blue box) [34], MCHR1 (yellow box) [25], PKA regulatory subunit RIIα (orange boxes) [5] and Semaphorin 4C (pink box) [52] are mapped. The N-terminal 100 amino acids of Norbin, which stimulate neurite outgrowth [11], are highlighted (green box). Palmitoylation of cysteines C3 and C4 is critical for endosomal localisation of Norbin in neuronal dendrites [10]. The alternative start codon for the splice variant (isoform-2, M18) [4] is highlighted above, as are known point mutations, including five of clinical relevance (bold). E433Q, R478E, W652R and P652L are found in patients with developmental delay, intellectual disability and epilepsy [50]. All four mutations impair Norbin-dependent neurite outgrowth. Additionally, W652R and P652L mutations in/near the mGlu_5_-binding region impair mGlu_5_ signalling, whereas E433Q and R478E do not [50]. P402* is found in a patient with frontotemporal dementia, and causes altered morphology of FUS-positive granules [54]. A687G is a mutation that was serendipitously found to block Norbin interaction with mGlu_5_ [14].

Norbin was identified as a GPCR adapter protein in a yeast two-hybrid screen for interactors of class A GPCR melanin-concentrating hormone receptor 1 (MCH_1_) [25]. Norbin was subsequently screened against other GPCRs and shown to interact with the C-terminal portions of 35 out of 55 GPCRs tested, including class C metabotropic glutamate receptors (mGluRs) [25,34,40]. The binding was mapped to membrane-proximal portions of the MCH_1_ and mGlu_5a_ tails and the Norbin C-terminus (Figure 6). Two adjacent Norbin-binding regions were identified in mGlu_5a_ [34], and recently an ostensibly innocuous A687G Norbin point mutation was shown to block mGlu_5_ binding [14] (Figure 6). However, Norbin binding to GPCRs is unpredictable, as there is no sequence homology ([34,40]; and unpublished observations), so Norbin is presumed to confer binding through its secondary structure.

### Control of GPCR trafficking by Norbin

Commonly, Norbin regulates the steady-state cell surface levels of GPCRs it interacts with. For example, mGlu_5_ cell surface localisation was elevated upon Norbin expression in N2a cells, except with Norbin-binding deficient mGlu_5_ mutants [34]. Knock-down of Norbin in rodent neurons reduced the cell surface localisation of mGlu_5_ and mGlu_1_ [14,34]. Similarly, more MCH_1_ localised at the surface of HEK293 cells expressing Norbin [25]. In contrast, the surface localisation of several class A GPCRs, including CXCR4 and C5a receptor (C5a_1_), was increased in Ncdn^Δmye^ mouse neutrophils, whereas CXCR1, which does not interact, localised normally [24,40]. The total cellular levels of GPCRs are unaffected ([34,40]; and unpublished observations)), so it is unlikely that Norbin regulates GPCR biosynthesis or degradation.

Norbin was assessed by several groups for its involvement in the agonist-induced internalisation of GPCRs. Initial reports suggested no role in the MCH-stimulated internalisation of MCH_1_ in HEK293 cells, with normal agonist-concentration dependence and kinetics [25]. However, Ojha et al. [14] showed that Norbin depletion in cortical and hippocampal neurons abrogates the agonist-induced internalisation of mGlu_1_ and mGlu_5_, rescued by shRNA-resistant Norbin. How Norbin regulates agonist-induced GPCR internalisation is under investigation, possibly by scaffolding the internalisation machinery or by inhibiting negative regulators. Norbin competes with periplakin (another promiscuous GPCR adapter) for binding to MCH_1_ [40], and it competes with calmodulin (involved in the agonist-induced internalisation of certain GPCRs) [41,42] for binding to mGlu_5_. [34]. In any event, Norbin likely uses distinct mechanisms to influence steady-state and agonist-induced GPCR trafficking. Norbin might regulate the constitutive internalisation of GPCRs, which often occurs at significant level [43–46], or the recycling of GPCRs from endosomes back to the plasma membrane. These processes warrant future investigation.

### Control of GPCR signalling pathways by Norbin

#### Norbin controls the activation of heterotrimeric G proteins

Norbin regulates GPCR signalling through the associated heterotrimeric G protein alpha subunits (Gα). Norbin expression in HEK293 cells reduces the activation (GTP-loading) of Gα_o_ and Gα_11_ upon stimulation of MCH_1_ with MCH [25]. It is unknown whether Norbin can affect GPCR signalling also through the Gβγ subunits, or independently of the heterotrimeric G protein altogether. However, it is clear that Norbin influences several pathways downstream of GPCRs, positively or negatively. Furthermore, Norbin can control GPCR signalling separately from GPCR trafficking. Firstly, many of its effects on GPCR signalling pathways are measurable within seconds, too fast for trafficking events. Secondly, for MCH_1_, Norbin was shown to control downstream Ca^2+^ signalling without obviously affecting MCH_1_ trafficking [25].

#### Norbin controls Ca^2+^, IP_3_ and CaMKII signalling downstream of GPCRs

One common pathway downstream of GPCRs is the activation of phospholipase C, which generates inositol 1,4,5-trisphosphate (IP_3_), leading to Ca^2+^ release from intracellular stores. Ca^2+^ mobilisation in response to MCH stimulation of MCH_1_ was reduced upon Norbin expression in HEK293 cells [25]. Similarly, stimulation of orexin-1 receptor (OX_1_) or thromboxane receptor (TP), which bind Norbin, also reduced Ca^2+^ release upon expression of Norbin [25,40]. In contrast, HEK293 cells expressing Norbin showed raised IP_3_ accumulation upon mGlu_5_ stimulation with the group 1 mGluR agonist l-quisqualic acid [34]. Stimulation with mGlu_5_ agonist 3,5-dihydroxyphenylglycine (DHPG) also caused significantly more Ca^2+^ oscillations in the presence of Norbin, except when mGlu_5_ mutants defective in Norbin binding were used [34]. Therefore, regulation of Ca^2+^ signalling requires direct interaction between Norbin and GPCR. Accordingly, Ca^2+^ levels were normal upon stimulation of the histamine H1 receptor (H1), which does not interact with Norbin [40].

Ca^2+^ binds numerous target proteins, including calmodulin, which results in the activation of Ca^2+^/calmodulin-dependent protein kinase II (CaMKII). CaMKII can signal downstream of mGlu_5_, among other receptors, and normal CamKII activity is essential for spatial learning [47–49]. Mice with neuronal Norbin deficiency showed increased CaMKII activity in hippocampal lysates and impaired spatial learning [32]. It remains to be investigated if Norbin regulation of CaMKII occurs downstream of GPCR signalling.

#### Norbin controls ERK signalling downstream of GPCRs

Stimulation of GPCRs commonly leads to ERK1/2 activation. However, Norbin did not affect ERK activity upon stimulation of MCH_1_ or histamine receptor H1, despite regulating Gα activity and Ca^2+^ mobilisation in these contexts [40]. In contrast, ERK activation upon DHPG stimulation of mGlu_5_ was dependent on Norbin in HEK293 cells [34] and SH-SY5Y cells [50]. Interestingly, ERK activation was lost when two Norbin variants with SNPs near or within the mGlu_5_-binding region, identified in patients with intellectual disability, were expressed rather than wild-type Norbin [50], suggesting that Norbin promotes ERK signalling downstream of mGlu_5_. This is another example of separate roles of Norbin in GPCR trafficking and signalling. ERK activity downstream of mGlu_5_ is increased by Norbin, despite Norbin promoting the agonist-stimulated internalisation of mGlu_5_ and less receptor remaining on the cell surface. There are also examples of Norbin inhibiting ERK signalling downstream of GPCRs. Stimulation of Norbin-deficient mouse neutrophils with f-Met-Leu-Phe (fMLP), ligand of the GPCRs formyl peptide receptor (FPR) 1 and 2, caused elevated ERK activation compared with wild-type, suggesting that Norbin limits FPR-dependent ERK activity [24].

ERK is best known for controlling proliferation. Norbin is required for the proliferation and maturation of neuronal precursors during adult neurogenesis [33], and we have evidence that Norbin is required for the growth, survival and cell cycle progression of PC12 cells (manuscript under revision). It is conceivable that Norbin dysregulation contributes to diseases of deregulated proliferation, such as cancer, through its role in ERK signalling. Furthermore, ERK activity is also required for ROS production, and we showed that the elevated ROS production in Ncdn^Δmye^ neutrophils, which underlies their enhanced capacity to kill bacteria, can be reversed by ERK inhibitors [24], so the control of ERK activity also appears important for the immune functions of Norbin.

#### Norbin regulates Rac-GEFs and Rac downstream of GPCRs

We showed that Norbin binds the Rac-GEF P-Rex1 directly, at the PH domain, and that interaction between P-Rex1 and Norbin also occurs in cells [12]. Norbin and P-Rex1 promote each other's localisation at the plasma membrane, where P-Rex1 needs to be localised to activate Rac [12]. Norbin also directly stimulates the Rac-GEF activity of P-Rex1 *in vitro*, enhancing the limited constitutive Rac-GEF activity of P-Rex1, as well as its PIP_3_- and Gβγ-stimulated activities [12]. Norbin expression in HEK293 cells led to increased P-Rex1-dependent Rac activation in response to lysophosphatidic acid stimulation [12]. These results suggested Norbin positively regulates Rac-GEF function. Rac activity is required for changes in cell morphology, adhesion and migration, through the control of actomyosin cytoskeletal dynamics. Indeed, we showed Norbin promotes Rac-dependent endothelial cell morphologies upon stimulation with lysophosphatidic acid [12]. Others showed that Norbin controls the morphology and synaptic plasticity of neuronal cells [11,13,18,34], which are also Rac-dependent. Hence, it is conceivable that dysregulation of Rac-GEFs and Rac activity underlie Norbin-associated neurological disorders (see below).

Norbin can also act as a suppressor of Rac activity, as Ncdn^Δmye^ neutrophils have elevated Rac1 and Rac2 activities when stimulated with fMLP, independently of P-Rex1 [12,24]. Use of Rac inhibitors showed this Rac activity is required for the elevated ROS production in Ncdn^Δmye^ neutrophils which confers the increased capacity to kill bacteria [24]. Furthermore, we identified Vav1 as another Rac-GEF target of Norbin, as Vav1 activity was increased in Norbin-deficient neutrophils [24]. Thus, Norbin can both promote and limit Rac-GEF function and Rac activity, depending on context, and at least in part downstream of GPCR signalling.

### Norbin regulates protein kinase A

Protein kinase A (PKA) is a cAMP-dependent kinase regulated by multiple mechanisms, including binding of A-kinase anchoring proteins (AKAPs) [51]. AKAPs are scaffolds which confine PKA signalling to specific membrane microdomains or organelles through interactions with other proteins [51]. Norbin acts as an atypical AKAP by binding to the PKA regulatory subunit RIIα with nanomolar affinity [5]. Two separate locations on Norbin, aa 65–84 and 277–291, play a role in this interaction [5]. Association of Norbin with PKA within the cell presumably occurs at the plasma membrane, as it is required for the agonist-induced internalisation of mGlu_5_ in hippocampal neurons [14], for the recruitment of PKA to AMPA receptors (AMPARs), and for the mGluR-mediated endocytosis of AMPARs, important steps in group I mGluR-dependent synaptic plasticity [14]. Hence, the regulation of PKA by Norbin appears closely linked to GPCR trafficking, but it remains to be seen if it affects PKA activity and PKA signalling downstream of GPCRs.

### Norbin interactions with other proteins

Several other proteins bind Norbin, including Dia1, a Rho-GTPase effector crucial for actin cytoskeleton dynamics. Norbin was isolated with GST-Dia1 from mouse brain lysate, and interacts with Dia1 through its N-terminal 100 aa, binding to the FH3 domain of Dia1. Co-expression of Norbin aa 1–100 and Dia1 resulted in elevated neurite outgrowth, independently of the actin-polymerising activity of Dia1 [11]. Additionally, Norbin interacts with Semaphorin 4C (Sema4C), a transmembrane receptor for plexin in the central nervous system. Norbin was isolated with GST-Sema4C from mouse brain, and the proteins interact *in vitro* (Figure 6), as well as upon co-expression in HEK293 cells [52]. The functional consequences of this interaction have yet to be elucidated. Finally, Norbin was recently identified as an interactor of the lysosomal enzyme iduronate-2-sulfatase in mouse brain lysates using an affinity chromatography approach, but again any functional consequences remain unknown [53].

## Norbin dysregulation in disease

Norbin dysregulation is linked to neurological conditions. One recent study identified six patients displaying developmental delay, intellectual disability, and epilepsy, with four separate inherited or *de novo* missense variants in *NCDN* (Figure 6) [50]. In contrast to wild-type Norbin, these variants were unable to restore neurite-outgrowth defects in Norbin-deficient SH-SY5Y cells [50]. Two of the Norbin mutations fell within or adjacent to the mGlu_5_-binding region (Figure 6), and displayed defective mGlu_5_-dependent ERK signalling, whereas two variants with mutations more distant to the mGlu_5_-binding region did not [50].

Nicolas et al. [54] identified a *de novo* nonsense mutation in a patient with frontotemporal dementia, corresponding to a ∼30% reduction in Norbin expression in brain, and thus suspected haploinsufficiency (Figure 6). The disease subtype is associated with fused in sarcoma (FUS)-positive protein aggregates, and shRNA Norbin-depleted rat cortical neurons contained fewer but larger FUS-containing granules. Interestingly FUS depletion reduced Norbin expression, indicating a co-dependence speculated to contribute to disease pathophysiology [54].

Post-mortem examination of dorsolateral prefrontal cortex samples from schizophrenia patients showed significantly reduced Norbin expression [55]. To control for possible effects of drugs commonly prescribed for schizophrenia, Norbin levels were evaluated in rats treated with these drugs, and were normal [55].

Deregulation of Norbin is associated with bone and cartilage defects. In nucleus pulposus cells in the disks between vertebrae, loss of Norbin occurs during normal aging. Norbin-rich juvenile cells were proposed as future disk repair strategies [56]. Furthermore, a SNP in *NCDN* is linked to osteochondrosis in horses [57], and mice with heterozygous Norbin deletion show cartilage abnormalities reminiscent of chondrocyte proliferation and differentiation abnormalities [31].

Norbin autoantibodies are linked to various neurological conditions, including ataxia, dystonia, encephalopathy, cerebellar degeneration and Alzheimer's disease [58–65]. It is debated whether these autoantibodies contribute to the disease pathology, instead they were proposed to be symptomatic, as the result of a T-cell driven autoimmune response [62]. Accordingly, patients with cerebellar degeneration did not respond to antibody-depletion therapy, whereas long-term immunosuppressive treatment stabilised or improved their condition, despite Norbin antibody levels remaining unaltered [63]. Norbin protein was detected in patient plasma after ischemic stroke [66], suggesting that neuronal cell death associated with neurological conditions may cause cytosolic Norbin becoming available as an extracellular antigen. As mentioned above, several reports detected Norbin extracellularly, but it remains to be seen if this is relevant in autoantibody generation.

Spinal muscular atrophy (SMA) is caused by decreasing levels of survival motor neuron (SMN), a protein associated with mRNA transport. Norbin forms a complex with SMN, and SH-SY5Y cells depleted of SMN have fewer Norbin-containing cytoplasmic foci, whereas Norbin depletion increased SMN foci [15]. The authors speculated that the reduced SMN level seen in SMA may compromise Norbin localisation and function.

## Conclusions and future directions

Since Norbin was first discovered, it has been clear that this protein plays crucial roles in the nervous system. However, recent research has shown that there is much more to Norbin. Of particular interest is its emerging role as an immune suppressor. Further investigation of Norbin in the immune system are required, especially to interrogate Norbin deregulation in immune diseases. Preliminary analysis of the NCBI GEO Profiles database (ncbi.nlm.nih.gov/geoprofiles) suggests altered Norbin levels in macrophages and monocytes during various infectious and inflammatory diseases.

Current efforts are concentrated on the molecular mechanisms through which Norbin controls cell responses, particularly its control of GPCR trafficking and signalling. For example, roles of Norbin in constitutive GPCR internalisation and recycling are being investigated. Generally, more mutational analysis would be beneficial for investigating the consequences of Norbin interactions with proteins or lipids. Ideally, one would generate mutants that affect Norbin-dependent GPCR signalling without affecting GPCR trafficking, so that these roles can be differentiated.

The involvement of Norbin in human neurological diseases means therapeutic control of Norbin is desirable. However, Norbin is not a good drug target, because it is an intracellular adaptor protein that works through interaction with other proteins, which is notoriously hard to tackle. Instead, future work could investigate avenues of targeting Norbin-effector GPCRs, to manipulate Norbin indirectly. In diseases where Norbin expression or its GPCR interactions are affected, GPCR levels at the cell surface will be altered, so drugs to target these GPCRs may be beneficial.

## Perspectives

- Norbin is an essential protein which is conserved from plants to human, without any homologies to other proteins. It binds to numerous GPCRs, controlling their trafficking and signalling, through downstream pathways which remain incompletely understood but include Rac, ERK, and Ca^2+^.
- Norbin is well-known as an important regulator of neuronal function, but it was recently discovered that Norbin is also a suppressor of neutrophil-mediated innate immunity.
- Considering its critical roles and interesting modes of action, Norbin remains woefully understudied, so there is immense scope for important future discoveries. It would be of great interest to identify mechanisms of regulation, particularly any post-translational modifications that may acutely affect Norbin function.

## Abbreviations

AKAP: A-kinase anchoring protein
AMPAR: AMPA receptor
C5a1: C5a receptor C5a1
CaMKII: Ca^2+^/calmodulin-dependent protein kinase II
DHPG: 3,5-dihydroxyphenylglycine
fMLP: f-Met-Leu-Phe
FPR: formyl peptide receptor
FUS: fused in sarcoma
G protein: guanine nucleotide-binding protein
GEF: guanine-nucleotide exchange factor
GPCR: G protein-coupled receptor
H1: histamine H1 receptor
IP_3_: inositol 1,4,5- trisphosphate
LTD: long-term depression
LTP: long-term potentiation
MCH: melanin-concentrating hormone
MCH_1_: melanin-concentrating hormone receptor 1
mGlu_1_: metabotropic glutamate receptor 1
mGlu_5_: metabotropic glutamate receptor 5
mGluR: metabotropic glutamate receptor
NET: neutrophil extracellular trap
NFAT: nuclear factor of activated T cells
Norbin: neurite-outgrowth-related protein from the rat brain (Neurochondrin, NCDN)
OX_1_: orexin-1 receptor
PKA: protein kinase A
P-Rex1: PIP_3_-dependent Rac exchanger 1
ROS: reactive oxygen species
Sema4C: Semaphorin 4C
SMA: spinal muscular atrophy
SMN: survival motor neuron
SNP: single nucleotide polymorphism
TP: thromboxane receptor
